# Possible Effect of Binaural Beat Combined With Autonomous Sensory Meridian Response for Inducing Sleep

**DOI:** 10.3389/fnhum.2019.00425

**Published:** 2019-12-02

**Authors:** Minji Lee, Chae-Bin Song, Gi-Hwan Shin, Seong-Whan Lee

**Affiliations:** ^1^Department of Brain and Cognitive Engineering, Korea University, Seoul, South Korea; ^2^Department of Artificial Intelligence, Korea University, Seoul, South Korea

**Keywords:** sleep, theta wave, binaural beat, autonomous sensory meridian response, electroencephalography

## Abstract

Sleep is important to maintain physical and cognitive functions in everyday life. However, the prevalence of sleep disorders is on the rise. One existing solution to this problem is to induce sleep using an auditory stimulus. When we listen to acoustic beats of two tones in each ear simultaneously, a binaural beat is generated which induces brain signals at a specific desired frequency. However, this auditory stimulus is uncomfortable for users to listen to induce sleep. To overcome this difficulty, we can exploit the feelings of calmness and relaxation that are induced by the perceptual phenomenon of autonomous sensory meridian response (ASMR). In this study, we proposed a novel auditory stimulus for inducing sleep. Specifically, we used a 6 Hz binaural beat corresponding to the center of the theta band (4–8 Hz), which is the frequency at which brain activity is entrained during non-rapid eye movement (NREM) in sleep stage 1. In addition, the “ASMR triggers” that cause ASMR were presented from natural sound as the sensory stimuli. In session 1, we combined two auditory stimuli (the 6 Hz binaural beat and ASMR triggers) at three-decibel ratios to find the optimal combination ratio. As a result, we determined that the combination of a 30:60 dB ratio of binaural beat to ASMR trigger is most effective for inducing theta power and psychological stability. In session 2, the effects of these combined stimuli (CS) were compared with an only binaural beat, only the ASMR trigger, or a sham condition. The combination stimulus retained the advantages of the binaural beat and resolved its shortcomings with the ASMR triggers, including psychological self-reports. Our findings indicate that the proposed auditory stimulus could induce the brain signals required for sleep, while simultaneously keeping the user in a psychologically comfortable state. This technology provides an important opportunity to develop a novel method for increasing the quality of sleep.

## Introduction

Sleep has a great impact on our health and is an important factor in determining the quality of life (Walker, 2008; Zhang et al., 2015; Weber and Dan, 2016; Lee et al., 2018). However, many researchers have reported that 25% of people feel that their sleep quality is not good (Soldatos et al., 2005; Lee M. et al., 2019). Since insufficient sleep is a common problem that leads to considerable health, social, and economic impacts (Hublin et al., 2001), a variety of methods have been developed to improve sleep quality (Besedovsky et al., 2017; Lee and Kim, 2017). Inducing sleep quickly is one way to improve the quality of sleep. Previous studies have applied transcranial direct current stimulation (D’Atri et al., 2016), transcranial magnetic stimulation (Massimini et al., 2007), and pharmacological approaches (Walsh et al., 2008; Feld et al., 2013) as methods for inducing sleep. However, these methods are impractical to users in real-life and occasionally have adverse effects (Bellesi et al., 2014; Santostasi et al., 2016). It has been suggested that the application of sensory stimuli, especially an auditory stimulus, provides a superior method for improving sleep quality compared to other means (Harmat et al., 2008; Chan et al., 2010; Bellesi et al., 2014; Besedovsky et al., 2017).

Electroencephalography (EEG) is a high resolution and low-cost tool that can measure very practical brain states (Lee et al., 2015, 2016; Kwak et al., 2017). Therefore, this tool is widely used to measure to observe the changed brain states for improving sleep quality. Brainwave entrainment is the use of an external rhythmic stimulus to generate frequency-dependent EEG responses that match the frequency of the stimuli (Huang and Charyton, 2008; Seifi Ala et al., 2018). Synchronized pulsing stimuli can induce a dominant EEG frequency that appears during a given cognitive state (Tang et al., 2015; da Silva Junior et al., 2019). One method for producing brainwave entrainment is the use of an auditory stimulus, called a binaural beat (Huang and Charyton, 2008). The binaural beat is an auditory illusion that is observed when oscillatory stimuli are delivered at two adjacent frequencies to each ear at the same time (Perez et al., 2019). The brain can recognize the frequency difference between the two sounds (Oster, 1973). This stimulus entrained steady-state auditory responses in the brain cortex at the beat frequency (Perez et al., 2019). Initially, the superior olivary complex in the brainstem receives a separate audible input from each ear. This beat is then recognized by neurons in the inferior colliculus (Schwarz and Taylor, 2005). The phase-locked neural activity of brainstem auditory pathways becomes consistent with the frequency-following response (Hink et al., 1980). The auditory evoked responses produced by the binaural beat can be recorded using EEG (Ozdamar et al., 2011). This method has recently been used to induce meditation and has correlated with thoughtful processes (Lavallee et al., 2011). A 3 Hz binaural beat was shown to induce delta activity and increase the duration of non-rapid eye movement (NREM) in sleep stage 3 (Jirakittayakorn and Wongsawat, 2018). In addition, a 6 Hz binaural beat produced meditative effects by inducing theta activity in the frontal and parietal-central regions (Jirakittayakorn and Wongsawat, 2017). The binaural beat at 15 Hz improved working memory by inducing beta activity in the brain (Beauchene et al., 2017). It was also possible to reduce the difficulty in initiating and maintaining sleep in patients with chronic insomnia by providing an audio-visual stimulus that gradually decreases from 8 to 1 Hz (Tang et al., 2015). However, it has also been reported that the repetitive and unnatural sound of the binaural beat could make people feel uncomfortable (Crespo et al., 2013). Some studies even claimed that the binaural beat could annoy people without inducing the desired mental states (Jirakittayakorn and Wongsawat, 2017). Exposure to binaural beats, which do not take into account the user’s current state, can even cause dizziness, as well as discomfort (Noor et al., 2013). This is probably related to the amygdala, a central structure associated with emotional processing. This area is connected to most sensory cortical areas and plays an important role in emotional modulation in the early stages of sensory information processing (Surakka et al., 1998). In addition, binaural beats seem to feel uncomfortable, in the sense that repeated auditory stimuli cause anxiety and depression (Watkins, 2008). This discomfort could, therefore, make some people reluctant to use binaural beats in the context of real-life. However, the relationship between binaural beats and subjective emotions is still not well studied, including the auditory pathway to binaural beats (Munro and Searchfield, 2019; Perez et al., 2019). Thus, further research regarding psychological effects related to binaural beats is needed.

To resolve the problems associated with the use of binaural beats, recent research has investigated the possibility of combining it with other sounds, such as piano music (Wiwatwongwana et al., 2016; Gantt et al., 2017). The binaural beat combined with music could provide relief for the cardiovascular stress response seen in military service members with post-deployment stress (Gantt et al., 2017). They also reported feeling less stressed and showed decreased low-frequency heart rate variability. Moreover, the anxiolytic effect of binaural beat music was investigated compared to plain music under general anesthesia (Wiwatwongwana et al., 2016). They also showed a significant reduction in heart rate and decreased operative anxiety in the patients who listened to the binaural beat combined with music. These beats are made more enjoyable for users to listen to stimuli if they incorporate natural sounds (Munro and Searchfield, 2019). Although the combined stimulation is effective for humans to compensate for the shortcomings of binaural beats, research on the various parameters (e.g., decibel, exposure duration, and frequency) is needed to optimize the combination of the two auditory stimuli (Chaieb and Fell, 2017). So far, few groups have applied combined stimuli (CS) in the context of sleep induction.

Autonomous sensory meridian response (ASMR) refers to sensory experiences such as psychological stability or pleasure in response to visual, auditory, tactile, olfactory, or cognitive stimuli (Barratt and Davis, 2015). Recently, many studies have reported that ASMR is an efficient way to relax people’s minds in academic and social circles (Cash et al., 2018; Smith et al., 2019b). In fact, many people use ASMR to relax their negative moods to lead to sleep, which is accompanied by a feeling of calmness and rest (Barratt et al., 2017). It also correlates with emotional and physiological states (Poerio et al., 2018; Smith et al., 2019a). Previous studies have reported that ASMR helps lead to sleep by relaxing mental states and reducing anxiety (Barratt and Davis, 2015; Lochte et al., 2018). However, these results are simple feelings based on subjective questionnaires. According to functional magnetic resonance imaging results, ASMR reduces salience and visual networks (Smith et al., 2019b) but increased activities related to sensation, motion, and attention (Smith et al., 2019a). Also, in EEG, alpha power decreased in the left frontal regions when listening to positive music but decreased in the right frontal regions when listening to negative music, respectively (Balasubramanian et al., 2018). In other words, the asymmetry of alpha activity in the prefrontal and frontal regions changes with emotion (Geethanjali et al., 2018; Bo et al., 2019). However, there is still a lack of objective evidence to support subjective emotions associated with ASMR based on neuroimaging studies.

In this study, we proposed a novel stimulus for inducing sleep, where we combined the binaural beat to entrain brainwaves at 6 Hz with ASMR. We hypothesized that only theta power increased with the binaural beats and combined stimulus. We also expected that optimal combination stimulus causes 6 Hz brainwaves due to binaural beats and makes a user comfortable and relaxed when using ASMR. Our hypothesis was also supported in that the dynamic natural sound had a higher spontaneous acceptance rate than static noise (Munro and Searchfield, 2019). In particular, we noted the change in the midline concerning sleep induction. Moreover, the change in theta power over the midline region was highly relevant, since it was directly related to the transition from wakefulness to sleep (Wright et al., 1995). There were two experimental sessions. In session 1, three auditory stimuli were presented in order to find the optimal combination ratio between the binaural beat and the ASMR trigger that initiates ASMR using natural sounds. In particular, the intensity levels of sounds are important when presenting auditory stimuli. The average hearing thresholds for normal adults is usually 20 dB at each ear (López-Caballero and Escera, 2017; Munro and Searchfield, 2019). Also, people feel quiet at sound levels of 30 dB, and 45 dB is recommended as background noise level (Kipnis et al., 2016). Sound levels between 60 and 80 dB are regarded as noisy, and sound levels more than 80 dB are harmful (Kipnis et al., 2016). In this regard, we have determined the ratio of combination between the two auditory stimuli. In session 2, we compared the effect of the optimally combined stimulus determined in session 1 with that of a sham condition (SHAM), binaural beats only, and ASMR triggers only. Questionnaires were conducted before and after the stimulation period to explore changes in the emotional states which sustain psychological stability. Our results suggest that combining the stimuli could relieve the discomfort of the binaural beat and have a stabilizing effect of ASMR for inducing sleep. These findings could help induce sleep quickly as a way to improve the quality of sleep.

## Materials and Methods

### Subjects

Fifteen healthy right-handed subjects (one female, average age of 24.9 ± 1.81 years) were included in this study. No subjects had any history of neurological disorders or hearing problems. The experiment was performed following the principles of the Declaration of Helsinki. This study was reviewed and approved by the Institutional Review Board at the Korea University (KUIRB-2019-0134-01), and informed consent was obtained from all subjects before the experiments.

### Proposed Auditory Stimuli

We used a 6 Hz binaural beat, which corresponds to the center of the theta band (4–8 Hz) that is the dominant frequency during NREM sleep stage 1 (Berry, 2018). In order to induce activity at 6 Hz, a 250 Hz carrier tone was delivered to the left ear (Pratt et al., 2010), and a 256 Hz offset tone was delivered to the right ear simultaneously using Gnaural software.

To offset the inconvenience of the binaural beat, we combined it with natural sounds that induce ASMR, because some ASMR triggers (e.g., whispering, tapping, and crisp sounds) can induce a tingling feeling or static-like sensation (Barratt and Davis, 2015). The five ASMR triggers (rain, sea waves, waterfall, forest, and river) were randomized. Supplementary Table 1 shows which stimuli were exposed to which subjects. The exact website links of the five sound are as follows: (i) rain: https://www.youtube.com/watch?v=euWoxhUkf_w, (ii) sea waves: https://www.youtube.com/watch?v=p76Vjvioypg&t=5861s, (iii) waterfalls: https://www.youtube.com/watch?v=73y1CqxVCeA, (iv) forest: https://www.youtube.com/watch?v=cvQLjfLw644&t=2752s, and (v) river: https://www.youtube.com/watch?v=nE_XAauwu1I&t=873s.

Three types of CS were created using MATLAB R2017a and presented using Psychtoolbox. When combining auditory stimuli, there were several parameters (e.g., decibel, exposure duration, and frequency) that may have impacted the effect of a combined stimulus. Here, we attempted to investigate the brain responses by controlling for all parameters except decibel ratios of the CS. In session 1, we tested three CS with different decibel ratios. A repetitive sinusoidal sound (i.e., binaural beat) played at a high volume can induce feelings of discomfort (Crespo et al., 2013). On the other hand, it is difficult to induce brainwaves within the desired frequency using low volume sounds. In this regard, we determined three combined ratios between binaural beats and ASMR triggers. In the binaural beats, the sound level was changed to 45 dB for the recommended background sound level, 30 dB for quiet sound level, and 20 dB for ensuring hearing thresholds in each condition. The sound intensity of ASMR triggers was fixed at 60 dB, which is a clear-sounding sound level, because the threshold is approximately 60 dB for auditory stimuli (Jirakittayakorn and Wongsawat, 2018). Specifically, the combined ratios are as follows: (i) CS1 – binaural beats:ASMR triggers = 45:60; (ii) CS2 – binaural beats:ASMR triggers = 30:60; and (iii) CS3 – binaural beats:ASMR triggers = 20:60. In session 2, the effects of the optimal CS determined in session 1 were compared to the SHAM condition, binaural beats only and ASMR triggers only. For the SHAM condition, a silent stimulus was used with earphones in each ear (Garcia-Molina et al., 2018; Jirakittayakorn and Wongsawat, 2018; Lustenberger et al., 2018). The volume of the ASMR trigger only was set to 60 dB, which has been previously determined to be a comfortable level (Stevens et al., 2003). In the case of 6 Hz binaural beat only, subjects were also exposed to 60 dB.

### Experimental Procedures

**FIGURE 1:**
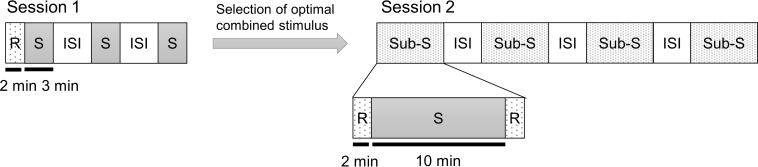
Experimental paradigm. The experiment consisted of two sessions. Session 1 was to determine the optimal decibel ratio for combining a binaural beat and an ASMR trigger. In session 2, the CS selected from session 1 was compared to the SHAM, BB only, and AT only condition. Each auditory stimulus is presented as a random order. Session 1: CS1 = 45:60 BB:AT ratio; CS2 = 30:60 BB:AT ratio; CS3 = 20:60 BB:AT ratio. Session 2: SHAM = sham condition, BB = binaural beats, AT = autonomous sensory meridian response triggers, CS = combined stimuli of BB and AT, S = stimulus, ISI = inter-stimulus interval, Sub-S = Sub session, R = resting state.

Figure 1 shows the experimental paradigms for session 1 and session 2. During all of the experiments, the subjects kept their eyes closed. Session 1 began with an assessment of emotional states using questionnaires. After responding to the questionnaires, they remained at resting state without hearing any stimulus for 2 min with his/her eyes closed as the baseline. The auditory stimuli were delivered through earphones for 3 min while subjects kept their eyes closed. A 2 min stimulation period is an acceptable timeframe to detect the effect of an auditory stimulus (Goodin et al., 2012). Therefore, we exposed the subject to the combined stimulus for 3 min to accurately induce the desired frequency. The three CS conditions were presented in a counterbalanced random order. The subject completed the questionnaires after listening to each CS. Inter-stimulus intervals of 5–10 min were provided between the stimulation periods, and the subjects were allowed to wake up sufficiently.

The aim of session 2 was to explore whether the CS could aid in inducing psychological stability and activation of brain signals at the target frequency compared to the SHAM, binaural beats only, and ASMR triggers only conditions. In other words, there were four sub-sessions (SHAM, binaural beats only, ASMR triggers only, and CS conditions) in session 2. The sub-session interval was set to 5–10 min, as with session 1, to minimize any effect of the previous stimulus to disturb the response of the upcoming stimulus. At the end of session 1, we selected the combined ratio that best-induced theta power for each individual and used it for session 2. In session 1, we focused on whether theta power occurs in each of the three CS conditions with different decibel rates, whereas session 2 investigated sleep induction and the continuous effects of auditory stimuli. Therefore, session 2 differed from session 1 in two respects. First, we presented an auditory stimulus for 10 min in session 2. This is because activity in all brain regions is enhanced by an auditory stimulus within 10 min of exposure (Jirakittayakorn and Wongsawat, 2017). Second, the resting states were measured before and after stimulation for 2 min in each sub-session. Subjects were similarly given 2 min of rest with their eyes closed without earphones on. In the many previous studies, resting states were maintained 2 min (Schwab et al., 2014; Sandler et al., 2016) or shorter (López-Caballero and Escera, 2017; Zhao et al., 2018). In addition, resting states before each stimulus were used as the baseline in the analysis. Each stimulus was assigned at random and counterbalanced across subjects. The subjects were also asked to complete questionnaires after listening to each stimulus.

We also investigated the changes in psychological stability before and after all auditory stimuli. The 32-item Brunel Mood Scale (BRUMS-32) was used (Lane and Jarrett, 2005). The original BRUMS is a 24-item mood scale based on the Profile of Mood States (Terry et al., 1999, 2003). The BRUMS-32 was formed by adding items that assess the subscales of “happy” and “calmness” (Terry et al., 1999). These questionnaires have eight factors, each with four mood descriptors (Supplementary Table 2). The factors are “anger,” “tension,” “depression,” “vigor,” “fatigue,” “confusion,” “happy,” and “calmness.” Subjects responded with a rating on the 5-point Liker scale, where “0” = “not at all,” “1” = “a little,” “2” = “moderately,” “3” = “quite a bit,” and “4” = “extremely.” Thus, the total score for one factor is a maximum of 16 (4 mood descriptors × 4 points).

### EEG Acquisition and Analysis

#### EEG Recording

The EEG data were recorded at a sampling rate of 500 Hz. We used an EEG amplifier (BrainAmp, Brain Product GmbH, Germany) with 19 Ag/AgCl electrodes using the 10-10 international system configuration. EEG data were referenced to the FCz electrode, and the ground channel was the AFz electrode.

#### EEG Data Analysis

All data analysis was performed using MATLAB R2017a with the OpenBMI toolbox (Lee M.H. et al., 2019) and BBCI toolbox (Blankertz et al., 2016). The EEG data were down-sampled to 250 Hz. A band-pass finite impulse response filter was applied between 0.5 and 50 Hz, as this filter is stable and simple (Correa et al., 2007). In addition, a notch filter was performed at 60 Hz for the elimination of power transmission lines (Olguin et al., 2005).

The fast Fourier transform was performed to convert from the time domain to the frequency domain for spectral analysis. We analyzed the changes of auditory stimuli in five frequency bands: delta (0.5–4 Hz), theta (4–8 Hz), alpha (8–13 Hz), beta (13–30 Hz), and gamma bands (30–50 Hz), as the spectral elements of the EEG signals, are normally divided into these frequencies (Jirakittayakorn and Wongsawat, 2018). However, we focused on the theta power, since we induced theta activity using the 6 Hz binaural beat. In addition, we investigated spatial changes in theta activity in seven brain regions: the prefrontal, frontal, central, temporal, parietal, occipital, and midline regions (Table 1). These areas were chosen because the brain areas associated with binaural beats are not uncertain (Munro and Searchfield, 2019; Perez et al., 2019). The power in frequency bands was computed in each channel and averaged in seven regions.

**TABLE 1:** Brain regions and their corresponding EEG channels.

**Brain regions**	**EEG channels**
Prefrontal	Fp1, Fp2, F7, F8
Frontal	F3, Fz, F4
Central	C3, Cz, C4
Temporal	T7, T8, P7, P8
Parietal	P3, Pz, P4
Occipital	O1, O2
Midline	Fz, Cz, Pz

In session 1, we used 6 Hz peak (desired frequency) over midline when selecting CS to be used in session 2. Because theta power of midline is directly related to transition to sleep (Wright et al., 1995). Additionally, we measured the laterality index (LI) to investigate the asymmetry in the alpha band related to emotion in session 2. This index was calculated by the following equation: LI = (L − R)/(L + R), where L and R represent the left and right hemispheres, respectively (Kikuchi et al., 2011). This value is between −1 and 1. Specifically, the positive value refers to left-hemisphere dominance, whereas the negative value refers to right-hemisphere dominance (Ito and Liew, 2016). We calculated LI over both the prefrontal and frontal regions that are related to emotions.

#### Statistical Analysis

We performed a paired *t*-test to investigate which frequency was induced after listening to each auditory stimulus. The paired *t*-test between before and after each stimulus was also applied to explore the induced theta power in the seven areas. We applied a one-way analysis of variance (ANOVA) to explore the spatial differences of theta power among stimuli. For *post hoc* analysis, paired *t*-tests were used with the Bonferroni correction. Similarly, we examined the changes in psychological stability for each item compared to baseline using paired *t*-tests. The differences in the BRUMS-32 scores among the stimuli were then compared using ANOVA and paired *t*-test for *post hoc* analysis at each psychological factor.

Additionally, in session 2, to examine spatial changes of resting states before and after auditory stimuli, theta power over 19 EEG electrodes was performed using two-way ANOVA (channel × the absence or presence of auditory stimuli). The paired *t*-tests with the Bonferroni correction were also performed about only spatial differences between before and after auditory stimuli in each channel for *post hoc* analysis. The paired *t*-test was also performed to investigate the difference in LI after each stimulus. The alpha level for all statistical significance was set at 0.05. The effect size was calculated as Cohen’s *d* and $\eta_{\text{p}}^{2}$ for paired *t*-test and ANOVA, respectively.

## Results

### Session 1: Optimal Combined Ratio Between Binaural Beat and ASMR Trigger

#### Brainwave Entrainment

In session 1, subjects were presented with three different binaural beats to ASMR triggers ratios: 45:60, 30:60, and 20:60. Table 2 shows statistical differences in power between the five frequency bands compared to baseline. Note the increase in power of the theta band, which was the target frequency we aimed to induce with the binaural beat. For the three CS conditions, other frequencies in the brain were not induced.

**TABLE 2:** Statistics of power differences between baseline and auditory stimuli in session 1.

		**Delta**	**Theta**	**Alpha**	**Beta**	**Gamma**
CS1	Power	−0.10 ± 1.06	0.12 ± 0.19	0.07 ± 0.41	0.03 ± 0.07	0.01 ± 0.05
	*df*	14	14	14	14	14
	*t*-value	−0.370	2.432	0.714	1.518	0.896
	*p*-value	0.716	*0.029*	0.486	0.151	0.385
	Cohen’s *d*	0.097	0.625	0.179	0.393	0.235
CS2	Power	0.08 ± 0.66	0.18 ± 0.20	0.22 ± 0.82	0.06 ± 0.23	0.01 ± 0.19
	*df*	14	14	14	14	14
	*t*-value	0.472	3.461	1.025	1.024	0.192
	*p*-value	0.643	*0.003*	0.322	0.322	0.849
	Cohen’s *d*	0.119	0.890	0.264	0.265	0.048
CS3	Power	−0.08 ± 0.96	0.13 ± 0.15	−0.01 ± 0.46	0.03 ± 0.12	0.02 ± 0.10
	*df*	14	14	14	14	14
	*t*-value	−0.332	3.266	−0.079	0.8555	0.972
	*p*-value	0.744	*0.005*	0.937	0.406	0.347
	Cohen’s *d*	0.088	0.843	0.023	0.230	0.249

*The changed of power, degree of freedom (*df*), *t* and *p*-values, and size effects (Cohen’s *d*) are presented. The *p*-values below 0.05 are highlighted in italics. CS1 = 45:60 BB:AT ratio; CS2 = 30:60 BB:AT ratio; CS3 = 20:60 BB:AT ratio. BB = binaural beat, AT = autonomous sensory meridian response trigger, CS = combined stimuli with BB and AT; power = mean ± standard deviation.*

**FIGURE 2:**
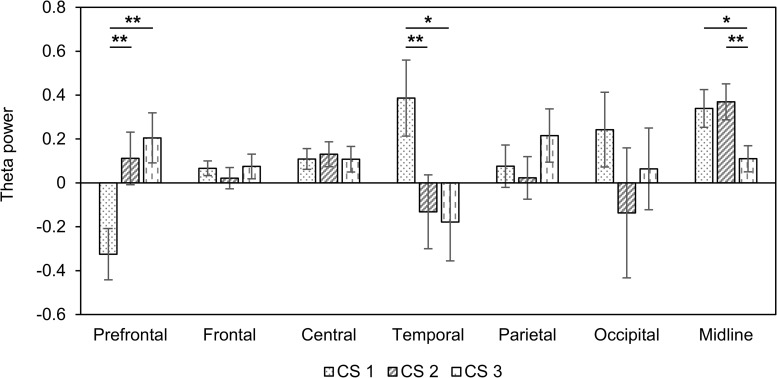
Changes in theta power compared to baseline in three combined stimuli conditions in session 1. Error bars show standard error. CS1 = 45:60 BB:AT ratio; CS2 = 30:60 BB:AT ratio, CS3 = 20:60 BB:AT ratio. BB = binaural beat, AT = autonomous sensory meridian response trigger, CS = combined stimuli with BB and AT. ^∗^<0.05 with no correction and ^∗∗^<0.05 with Bonferroni correction.

Next, we assessed changes in theta power in seven areas of the brain (Table 3). In the prefrontal region, there was a decreased theta power in only CS1. On the other hand, theta power increased in only CS1 in temporal regions. We observed the increased theta power in both CS1 and CS2 over the central and midline regions. Figure 2 shows changes in theta power caused by three CSs compared to baseline. There were statistical differences among three CSs in the prefrontal (*F*_(__2_, _42__)_ = 5.85, *p* = 0.005, $\eta_{\text{p}}^{2}$ = 0.217), temporal (*F*_(__2_, _42__)_ = 3.29, *p* = 0.047, $\eta_{\text{p}}^{2}$ = 0.135), and midline regions (*F*_(__2_, _42__)_ = 3.42, *p* = 0.042, $\eta_{\text{p}}^{2}$ = 0.140). In the prefrontal region, theta power in CS2 and CS3 was significantly higher than in CS1 (CS1 vs. CS2: *df* = 14, *t* = −3.577, *p* = 0.003, Cohen’s *d* = 0.923; CS1 vs. CS3: *df* = 14, *t* = −4.046, *p* = 0.001, Cohen’s *d* = 1.044), but in the temporal region, theta power in CS1 was significantly higher than in CS2 and CS3 (CS1 vs. CS2: *df* = 14, *t* = 3.982, *p* = 0.001, Cohen’s *d* = 1.028; CS1 vs. CS3: *df* = 14, *t* = 2.699, *p* = 0.017, Cohen’s *d* = 0.697). In the midline regions, theta power in CS1 and CS2 was significantly higher than in CS3 (CS1 vs. CS3: *df* = 14, *t* = 2.565, *p* = 0.022, Cohen’s *d* = 0.662; CS2 vs. CS3: *df* = 14, *t* = 3.430, *p* = 0.004, Cohen’s *d* = 0.885).

**TABLE 3:** Spatial changes before and after combined stimuli in session 1.

		**Prefrontal**	**Frontal**	**Central**	**Temporal**	**Parietal**	**Occipital**	**Midline**
CS1	Power	−0.33 ± 0.45	0.07 ± 0.13	0.11 ± 0.18	0.39 ± 0.67	0.08 ± 0.37	0.24 ± 0.66	0.34 ± 0.33
	*df*	14	14	14	14	14	14	14
	*t*-value	−2.780	1.993	2.312	2.235	0.785	1.422	3.944
	*p*-value	*0.014*	0.066	*0.036*	*0.042*	0.445	0.176	*0.001*
	Cohen’s *d*	0.717	0.515	0.597	0.577	0.202	0.367	1.018
CS2	Power	0.11 ± 0.47	0.02 ± 0.19	0.13 ± 0.22	−0.13 ± 0.65	0.02 ± 0.38	−0.14 ± 1.15	0.37 ± 0.32
	*df*	14	14	14	14	14	14	14
	*t*-value	0.929	0.440	2.299	−0.780	0.233	−0.461	4.507
	*p*-value	0.368	0.666	*0.037*	0.448	0.819	0.651	<*0.001*
	Cohen’s *d*	0.240	0.113	0.593	0.201	0.060	0.119	1.164
CS3	Power	0.21 ± 0.44	0.07 ± 0.22	0.11 ± 0.23	−0.18 ± 0.69	0.22 ± 0.47	0.06 ± 0.72	0.11 ± 0.23
	*df*	14	14	14	14	14	14	14
	*t*-value	1.796	1.324	1.858	−1.004	1.781	0.344	1.851
	*p*-value	0.094	0.206	0.084	0.332	0.096	0.736	0.085
	Cohen’s *d*	0.463	0.341	0.480	0.259	0.460	0.088	0.477

*The changed of power, degree of freedom (*df*), *t*- and *p*-values, and size effects (Cohen’s *d*) are presented. The *p*-values below 0.05 are highlighted in italics. CS1 = 45:60 BB:AT ratio; CS2 = 30:60 BB:AT ratio; CS3 = 20:60 BB:AT ratio. BB = binaural beat, AT = autonomous sensory meridian response trigger, CS = combined stimuli with BB and AT; power = mean ± standard deviation.*

#### Psychological Stability

We compared the BRUMS-32 scores before and after auditory stimuli to investigate the effect of inducing psychological stability (Table 4). Four negative emotional states (“anger,” “tension,” “depression,” and “confusion”) were decreased in all three CS conditions compared to baseline. However, the “vigor” scores (positive emotional state) were also reduced. There were no significant changes in the “fatigue” scores in all three CS conditions. Interestingly, the “happy” scores were only significantly increased in CS2. The scores in “calmness” were significantly reduced in CS1 compared to baseline.

**TABLE 4:** Statistical differences in the BRUMS-32 scores after each stimulus compared to baseline.

		**Anger**	**Tension**	**Depression**	**Vigor**	**Fatigue**	**Confusion**	**Happy**	**Calmness**
CS1	Score	−1.93 ± 1.91	−2.67 ± 3.18	−1.60 ± 1.55	−2.13 ± 3.27	−1.80 ± 3.90	−2.20 ± 1.86	−0.27 ± 2.71	−1.00 ± 1.65
	*df*	14	14	14	14	14	14	14	14
	*t*-value	−3.925	−3.250	−4.000	−2.526	−1.789	−4.582	−0.380	−2.350
	*p*-value	*0.001*	*0.005*	*0.001*	*0.024*	0.095	<*0.001*	0.709	*0.033*
	Cohen’s *d*	1.013	0.839	1.032	0.652	0.462	1.183	0.098	0.606
CS2	Score	−2.87 ± 2.53	−3.00 ± 2.42	−1.87 ± 1.51	−2.27 ± 2.66	−1.53 ± 3.87	−2.33 ± 1.76	1.33 ± 2.09	0.33 ± 1.72
	*df*	14	14	14	14	14	14	14	14
	*t*-value	−4.385	−4.800	−4.802	−3.302	−1.534	−5.136	2.467	0.751
	*p*-value	<*0.001*	< *0.001*	<*0.001*	*0.005*	0.147	<*0.001*	*0.027*	0.464
	Cohen’s *d*	1.132	1.239	1.239	0.852	0.396	1.326	0.636	0.193
CS3	Score	−2.73 ± 2.66	−3.00 ± 2.54	−2.07 ± 2.09	−1.87 ± 3.31	−1.33 ± 3.81	−1.67 ± 2.41	0.73 ± 2.60	0.93 ± 1.75
	*df*	14	14	14	14	14	14	14	14
	*t*-value	−3.982	−4.582	−3.836	−2.181	−1.355	−2.678	1.090	2.064
	*p*-value	*0.001*	<*0.001*	*0.002*	*0.046*	0.196	*0.018*	0.293	0.058
	Cohen’s *d*	1.028	1.183	0.990	0.563	0.349	0.691	0.281	0.532

*The changed of score, degree of freedom (*df*), *t* and *p*-values, and size effects (Cohen’s *d*) are presented. The *p*-values below 0.05 are highlighted in italics. CS1 = 45:60 BB:AT ratio; CS2 = 30:60 BB:AT ratio; CS3 = 20:60 BB:AT ratio. BB = binaural beat, AT = autonomous sensory meridian response trigger, CS = combined stimuli of BB and AT, score = mean ± standard deviation.*

**FIGURE 3:**
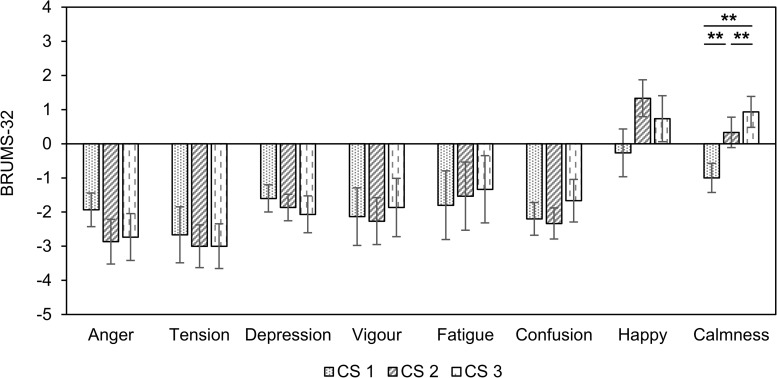
Changes in BRUMS-32 scores compared to baseline in three combined stimuli conditions in session 1. Error bars show standard errors. CS1 = 45:60 BB:AT ratio; CS2 = 30:60 BB:AT ratio; CS3 = 20:60 BB:AT ratio. BB = binaural beats, AT = autonomous sensory meridian response triggers, CS = combined stimuli of BB and AT. ^∗∗^<0.05 with Bonferroni correction.

We observed differences in the eight factors for emotional states in all three CS conditions (Figure 3). Only “calmness” indicated a significant difference among the three stimuli (*F*_(__2_, _42__)_ = 5.05, *p* < 0.010, $\eta_{\text{p}}^{2}$ = 0.193). The score for “calmness” in CS1 was significantly lower than in CS2 and CS3 (CS1 vs. CS2: *df* = 14, *t* = −3.005, *p* = 0.009, Cohen’s *d* = 0.775; CS1 vs. CS3: *df* = 14, *t* = −3.780, *p* = 0.002, Cohen’s *d* = 0.975). The “calmness” was also significantly higher in CS3 than in CS2 (*df* = 14, *t* = −2.806, *p* = 0.014, Cohen’s *d* = 0.724). There were no significant differences between the three CS conditions in the seven factors.

As a result, we observed the individual changes in 6 Hz peak over midline area and psychological stability for the three combined auditory stimuli, respectively. All subjects had the greatest increase in 6 Hz peak in CS2 compared to CS1 and CS3. However, in the case of Sub02, Sub03, Sub06, Sub08, the 6 Hz peak of CS2 was slightly increased compared to CS3. For Sub14, the 6 Hz peak of CS2 was slightly higher than CS1 (Supplementary Table 3). Consequently, we decided that CS2 was a more appropriate ratio for the consideration of psychological stability (Supplementary Figure 1). As a result, CS2 was selected as the optimal ratio between binaural beats and ASMR triggers for all subjects and was used for all experiments in session 2.

### Session 2: The Effect of Combined Stimuli for Inducing Sleep

#### Brainwave Entrainment

As a result of session 1, CS2 was chosen as the optimal ratio for combining the binaural beat and ASMR trigger. In session 2, subjects were presented with four auditory stimuli (SHAM, the binaural beat, ASMR trigger, and CS2). Table 5 shows the statistical results for inducing each frequency power after each auditory stimuli. We observed the induction of theta power in the binaural beat, ASMR trigger, and CS. Alpha power was significantly decreased with all auditory stimuli except for the SHAM condition. Additionally, beta power was significantly reduced in only the CS condition. No frequency changes were induced with the SHAM condition.

**TABLE 5:** Statistical differences in power between baseline and auditory stimulus in session 2.

		**Delta**	**Theta**	**Alpha**	**Beta**	**Gamma**
SHAM	Power	−0.05 ± 1.62	0.07 ± 0.97	−0.59 ± 1.06	−0.05 ± 0.20	0.00 ± 0.29
	*df*	14	14	14	14	14
	*t*-value	−0.131	0.286	−2.143	−1.005	−0.092
	*p*-value	0.897	0.778	0.051	0.331	0.927
	Cohen’s *d*	0.033	0.074	0.553	0.260	0.026
BB	Power	0.25 ± 0.80	0.40 ± 0.59	−0.96 ± 0.86	−0.02 ± 0.32	0.13 ± 0.29
	*df*	14	14	14	14	14
	*t*-value	1.888	2.603	−4.340	−0.246	1.672
	*p*-value	0.254	*0.020*	<*0.001*	0.808	0.116
	Cohen’s *d*	0.307	0.672	1.116	0.065	0.416
AT	Power	0.01 ± 2.01	0.28 ± 0.51	−1.32 ± 0.96	0.04 ± 0.20	0.08 ± 0.23
	*df*	14	14	14	14	14
	*t*-value	0.017	2.093	−5.347	0.756	1.308
	*p*-value	0.986	0.055	<*0.001*	0.461	0.211
	Cohen’s *d*	0.005	0.538	1.381	0.192	0.326
CS	Power	0.32 ± 1.30	0.48 ± 0.74	−1.39 ± 1.19	−0.09 ± 0.10	−0.02 ± 0.21
	*df*	14	14	14	14	14
	*t*-value	0.943	2.516	−4.539	−3.432	−0.318
	*p*-value	0.361	*0.024*	<*0.001*	*0.004*	0.754
	Cohen’s *d*	0.244	0.650	1.176	0.884	0.076

*The changed of power, degree of freedom (*df*), *t* and *p*-values, and size effects (Cohen’s *d*) are presented. The *p*-values below 0.05 are highlighted in italics. SHAM = sham condition, BB = binaural beat, AT = autonomous sensory meridian response trigger, CS = combined stimuli of BB and AT at the ratio of 30:60 dB; power = mean ± standard deviation.*

**FIGURE 4:**
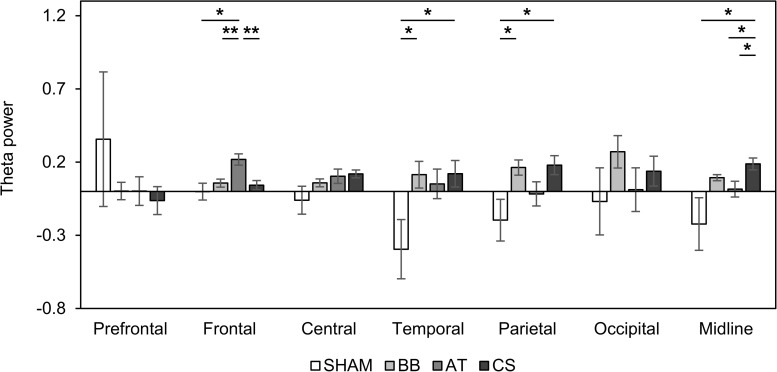
Changes in absolute theta power compared to baseline with four auditory stimuli in session 2. Error bars show standard errors. SHAM = sham condition, BB = binaural beats, AT = autonomous sensory meridian response triggers, CS = combined stimuli of 30:60 ratio between BB and AT. ^∗^<0.05 with no correction, ^∗∗^<0.05 with Bonferroni correction.

We examined spatial changes in theta power in the SHAM, the binaural beat, ASMR trigger, and CS conditions. In the prefrontal region, there was no significant change in theta power in all four conditions. The theta power increased in only the ASMR triggers in the frontal region, in only the CS in the central region, and in only the binaural beat in the occipital region. Finally, the theta power in both binaural beats and CS was significantly increased in the parietal and midline regions (Table 6). In addition, we investigated the differences in theta power between each condition in seven regions (Figure 4). As a result of ANOVA, there were significant differences in four regions (frontal region: *F*_(__3_, _56__)_ = 5.70, *p* = 0.002, $\eta_{\text{p}}^{2}$ = 0.234; temporal region: *F*_(__3_, _56__)_ = 3.61, *p* = 0.019, $\eta_{\text{p}}^{2}$ = 0.162; parietal region: *F*_(__3_, _56__)_ = 3.66, *p* = 0.018, $\eta_{\text{p}}^{2}$ = 0.163; midline region: *F*_(__3_, _56__)_ = 3.31, *p* = 0.027, $\eta_{\text{p}}^{2}$ = 0.150). Theta power with ASMR trigger was significantly higher than with SHAM, the binaural beat, and CS over the frontal region (SHAM vs. ASMR trigger: *df* = 14, *t* = −2.704, *p* = 0.017, Cohen’s *d* = 0.698; binaural beat vs. ASMR trigger: *df* = 14, *t* = −3.439, *p* = 0.004, Cohen’s *d* = 0.888; ASMR trigger vs. CS: *df* = 14, *t* = −3.704, *p* = 0.002, Cohen’s *d* = 0.887). In the temporal and parietal regions, theta power with the binaural beat and CS was significantly higher than with SHAM (temporal region: SHAM vs. BB: *df* = 14, *t* = −2.193, *p* = 0.045, Cohen’s *d* = 0.566; SHAM vs. CS: *df* = 14, *t* = −2.270, *p* = 0.039, Cohen’s *d* = 0.586; parietal region: SHAM vs. BB: *df* = 14, *t* = −2.150, *p* = 0.049, Cohen’s *d* = 0.555; SHAM vs. CS: *df* = 14, *t* = −2.297, *p* = 0.037, Cohen’s *d* = 0.593). Finally, in the midline region, theta power with CS was significantly higher than the other three conditions (SHAM vs. CS: *df* = 14, *t* = −2.415, *p* = 0.030, Cohen’s *d* = 0.623; binaural beat vs. CS: *df* = 14, *t* = −2.635, *p* = 0.019, Cohen’s *d* = 0.680 ASMR trigger vs. CS: *df* = 14, *t* = −2.417, *p* = 0.029, Cohen’s *d* = 0.624).

**TABLE 6:** Spatial changes before and after four auditory stimuli in session 2.

		**Prefrontal**	**Frontal**	**Central**	**Temporal**	**Parietal**	**Occipital**	**Midline**
SHAM	Power	0.36 ± 1.78	0.00 ± 0.22	−0.06 ± 0.37	−0.39 ± 0.78	−0.20 ± 0.55	−0.07 ± 0.89	−0.22 ± 0.70
	*df*	14	14	14	14	14	14	14
	*t*-value	0.774	−0.025	−0.635	−1.951	−1.378	−0.300	−1.237
	*p*-value	0.451	0.980	0.535	0.071	0.189	0.768	0.236
	Cohen’s *d*	0.199	0.006	0.164	0.503	0.355	0.077	0.319
BB	Power	0.00 ± 0.23	0.06 ± 0.11	0.06 ± 0.10	0.11 ± 0.35	0.16 ± 0.20	0.27 ± 0.43	0.09 ± 0.08
	*df*	14	14	14	14	14	14	14
	*t*-value	0.042	2.066	2.159	1.254	3.153	2.446	4.577
	*p*-value	0.966	0.057	0.048	0.230	*0.007*	*0.028*	<*0.001*
	Cohen’s *d*	0.010	0.533	0.558	0.323	0.814	0.631	1.181
AT	Power	0.00 ± 0.38	0.22 ± 0.15	0.10 ± 0.19	0.05 ± 0.39	−0.02 ± 0.32	0.01 ± 0.58	0.02 ± 0.21
	*df*	14	14	14	14	14	14	14
	*t*-value	0.025	5.656	2.099	0.507	−0.205	0.079	0.290
	*p*-value	0.979	<*0.001*	0.054	0.619	0.840	0.937	0.776
	Cohen’s *d*	0.006	1.460	0.542	0.131	0.053	0.020	0.074
CS	Power	−0.06 ± 0.37	0.04 ± 0.12	0.12 ± 0.10	0.12 ± 0.35	0.18 ± 0.25	0.14 ± 0.39	0.19 ± 0.15
	*df*	14	14	14	14	14	14	14
	*t*-value	−0.656	1.396	4.422	1.341	2.758	1.354	4.710
	*p*-value	0.522	0.184	<*0.001*	0.201	*0.015*	0.197	<*0.001*
	Cohen’s *d*	0.169	0.360	1.142	0.346	0.712	0.349	1.216

*The changed of power, degree of freedom (*df*), *t* and *p*-values, and size effects (Cohen’s *d*) are presented. The *p*-values below 0.05 are highlighted in italics. SHAM = sham condition, BB = binaural beat, AT = autonomous sensory meridian response trigger, CS = combined stimuli of BB and AT at the ratio of 30:60 dB; power = mean ± standard deviation.*

**FIGURE 5:**
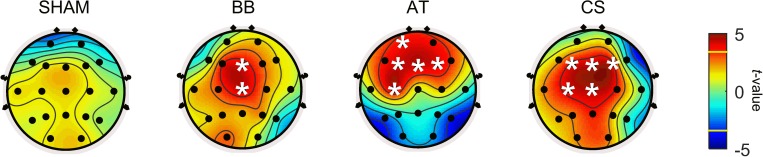
Statistical results between before and after the stimulation period for SHAM, BB, AT, and CS in session 2. The statistical differences of theta power before and after four auditory stimuli were calculated. A white asterisk indicates an electrode that is significantly different before compared to after stimulation (*p* < 0.05 with Bonferroni correction). In the color bar, the yellow line marks the *t*-value of the significant level. SHAM = sham condition, BB = binaural beats, AT = autonomous sensory meridian response triggers, CS = combined stimuli of BB and AT at the ratio of 30:60 dB.

In addition, theta power in the resting state was explored to investigate local changes before and after each stimulus. Figure 5 depicts the brain topography of theta power before and after the stimulation period for the SHAM, binaural beat, ASMR trigger, and CS conditions. Table 7 shows the statistical results of the two-way ANOVA. With SHAM, there was no change in theta power between pre- and post-stimulation. With the binaural beat, theta power increased significantly in the midline regions. In addition, power in the ASMR trigger was significantly increased in the prefrontal and frontal region after stimulation. With CS, theta power increased significantly in the frontal, central, and midline regions.

**TABLE 7:** Results of two-way ANOVA in spatial differences before and after four auditory stimuli.

**Type of auditory**	**Source**	***df***	***F*-value**	***p*-value**	**$\eta_{\text{p}}^{2}$**
SHAM	Channel	18	3.53	<*0.001*	0.106
	Stimulation	1	0.41	0.522	<0.001
	Channel × stimulation	18	0.08	0.999	0.002
BB	Channel	18	47.14	<*0.001*	0.614
	Stimulation	1	44.00	<*0.001*	0.076
	Channel × stimulation	18	1.53	0.073	0.049
AT	Channel	18	13.74	<*0.001*	0.317
	Stimulation	1	8.52	*0.003*	0.015
	Channel × stimulation	18	5.39	<*0.001*	0.154
CS	Channel	18	35.92	<*0.001*	0.548
	Stimulation	1	71.50	<*0.001*	0.118
	Channel × stimulation	18	2.75	<*0.001*	0.085

*The channel factor includes 19 electrode channels as spatial information, whereas the stimulation factor indicates the before and after auditory stimuli. The degree of freedom (*df*), *F* and *p*-values, and size effects ($\eta_{p}^{2}$) are presented. The *p*-values below 0.05 are highlighted in italics. SHAM = sham condition, BB = binaural beat, AT = autonomous sensory meridian response trigger, CS = combined stimuli of BB and AT at the ratio of 30:60 dB.*

We observed the alpha LI in the prefrontal and frontal regions to investigate the changes in emotions. In all stimuli, the LI had no change between pre- and post-stimulus (SHAM: *df* = 14, *t* = −1.406, *p* = 0.181, Cohen’s *d* = 0.362; binaural beat: *df* = 14, *t* = −1.941, *p* = 0.072, Cohen’s *d* = 0.503; ASMR trigger: *df* = 14, *t* = 1.177, *p* = 0.258, Cohen’s *d* = 0.305; CS: *df* = 14, *t* = −0.628, *p* = 0.540, Cohen’s *d* = 0.160) (Supplementary Figure 2). It also showed an LI near zero in all stimuli; therefore, there was little difference between the left and right hemispheres.

#### Psychological Stability

We investigated the psychological stability after four stimuli compared to baseline (Table 8). The “anger” score was statistically increased in the binaural beat but decreased in CS conditions. The “tension” scores were significantly decreased with both the ASMR triggers and CS conditions. The “depression” scores were not significantly changed, but the “vigor” scores decreased with all conditions. The “fatigue” scores showed significant increases with SHAM and CS conditions. The “confusion” scores were only statistically decreased with the ASMR triggers. The “happy” scores were significantly decreased with SHAM. Finally, the “calmness” score was decreased with the binaural beat but significantly increased with the ASMR triggers and CS conditions.

**TABLE 8:** Statistical differences in the BRUMS-32 scores with each stimulus compared to baseline.

		**Anger**	**Tension**	**Depression**	**Vigor**	**Fatigue**	**Confusion**	**Happy**	**Calmness**
SHAM	Score	0.73 ± 1.49	0.53 ± 1.41	−0.47 ± 1.30	−3.33 ± 3.48	2.73 ± 2.55	0.53 ± 1.60	−2.53 ± 2.61	−0.73 ± 2.25
	*df*	14	14	14	14	14	14	14	14
	*t*-value	1.910	1.467	−1.388	−3.712	4.153	1.292	−3.450	−1.261
	*p*-value	0.077	0.164	0.187	*0.002*	*0.001*	0.217	*0.002*	0.228
	Cohen’s *d*	0.493	0.378	0.358	0.958	1.072	0.333	0.890	0.325
BB	Score	1.40 ± 2.06	−1.53 ± 3.60	−0.80 ± 2.91	−4.20 ± 3.41	1.47 ± 2.77	−1.13 ± 2.45	−1.87 ± 3.38	−1.40 ± 2.44
	*df*	14	14	14	14	14	14	14	14
	*t*-value	2.627	−1.648	−1.065	−4.776	2.047	−1.794	−2.140	−2.218
	*p*-value	*0.020*	0.122	0.305	<*0.001*	0.060	0.094	0.051	*0.044*
	Cohen’s *d*	0.678	0.425	0.275	1.233	0.528	0.463	0.552	0.572
AT	Score	−1.07 ± 2.69	−1.93 ± 2.94	−1.33 ± 2.66	−4.27 ± 3.06	1.67 ± 3.27	−1.13 ± 1.92	−0.20 ± 2.68	1.07 ± 1.75
	*df*	14	14	14	14	14	14	14	14
	*t*-value	−1.538	−2.547	−1.938	−5.403	1.976	−2.283	−0.289	2.359
	*p*-value	0.146	*0.023*	0.073	<*0.001*	0.068	*0.039*	0.777	*0.033*
	Cohen’s *d*	0.397	0.657	0.500	1.395	0.510	0.589	0.074	0.609
CS	Score	−1.87 ± 1.64	−2.00 ± 1.96	−0.80 ± 1.82	−1.80 ± 3.17	1.67 ± 2.85	−1.00 ± 2.24	0.07 ± 2.28	1.87 ± 2.75
	*df*	14	14	14	14	14	14	14	14
	*t*-value	−4.403	−3.944	−1.701	−2.201	2.268	−1.732	0.113	2.630
	*p*-value	*0.001*	*0.001*	0.111	*0.045*	*0.040*	0.105	0.912	*0.020*
	Cohen’s *d*	1.137	1.018	0.439	0.568	0.585	0.447	0.029	0.679

*The changed of score, degree of freedom (*df*), *t* and *p*-values, and size effects (Cohen’s *d*) are presented. The *p*-values below 0.05 are highlighted in italics. SHAM = sham condition, BB = binaural beat, AT = autonomous sensory meridian response trigger, CS = combined stimuli of BB and AT at the ratio of 30:60 dB; scores = mean ± standard deviation.*

**FIGURE 6:**
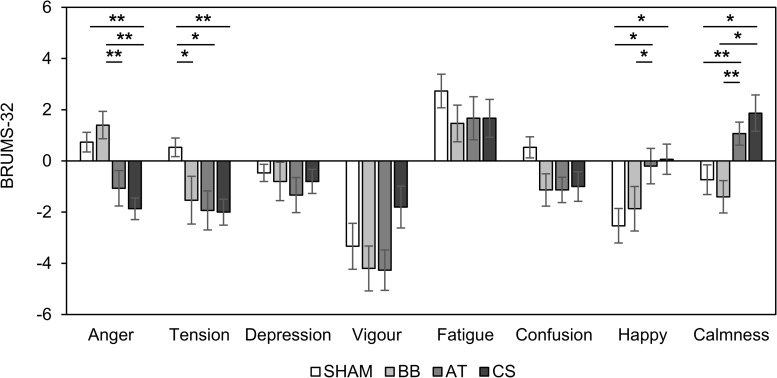
Changes in BRUMS-32 scores with four stimuli compared to baseline in session 2. Error bars show standard errors. SHAM = sham condition, BB = binaural beats, AT = autonomous sensory meridian response triggers, CS = combined stimuli of BB and AT at the ratio of 30:60 dB. ^∗^<0.05 with no correction, ^∗∗^<0.05 with Bonferroni correction.

Figure 6 shows the changes in BRUMS-32 scores with the four stimuli compared to baseline. There were statistical differences in four scores (“anger”: *F*_(__3_, _56__)_ = 8.50, *p* < 0.001, $\eta_{\text{p}}^{2}$ = 0.312; “tension”: *F*_(__3_, _56__)_ = 3.12, *p* = 0.033, $\eta_{\text{p}}^{2}$ = 0.143; “happy”: *F*_(__3_, _56__)_ = 2.87, *p* = 0.044, $\eta_{\text{p}}^{2}$ = 0.133; “calmness”: *F*_(__3_, _56__)_ = 6.43, *p* < 0.001, $\eta_{\text{p}}^{2}$ = 0.256). The BRUMS-32 scores for “anger” were significantly lower with CS than with SHAM and the binaural beat conditions (SHAM vs. CS: *df* = 14, *t* = 5.349, *p* = 0.022, Cohen’s *d* = 1.381; binaural beat vs. CS: *df* = 14, *t* = 4.304, *p* < 0.001, Cohen’s *d* = 1.111), and lower with ASMR trigger compared to the binaural beat binaural beat vs. ASMR trigger: *df* = 14, *t* = 3.953, *p* = 0.001, Cohen’s *d* = 1.020). The “tension” scores were significantly decreased in binaural beat, ASMR trigger, and CS conditions compared to SHAM (SHAM vs. binaural beat: *df* = 14, *t* = 2.251, *p* = 0.040, Cohen’s *d* = 0.581; SHAM vs. ASMR trigger: *df* = 14, *t* = 2.921, *p* = 0.011, Cohen’s *d* = 0.754; SHAM vs. CS: *df* = 14, *t* = 4.598, *p* < 0.001, Cohen’s *d* = 1.187). The “happy” scores showed the opposite trends compared to “tension” (SHAM vs. ASMR trigger: *df* = 14, *t* = −2.414, *p* = 0.030, Cohen’s *d* = 0.623; SHAM vs. CS: *df* = 14, *t* = −2.793, *p* = 0.014, Cohen’s *d* = 0.721; binaural beat vs. ASMR trigger: *df* = 14, *t* = −2.174, *p* = 0.047, Cohen’s *d* = 0.561). Finally, the “calmness” scores with ASMR triggers and CS conditions were significantly higher than with the SHAM and binaural beat conditions, respectively (SHAM vs. ASMR trigger: *df* = 14, *t* = −3.108, *p* = 0.007, Cohen’s *d* = 0.802; binaural beat vs. ASMR trigger: *df* = 14, *t* = −5.819, *p* < 0.001, Cohen’s *d* = 1.502; SHAM vs. CS: *df* = 14, *t* = −2.963, *p* = 0.010, Cohen’s *d* = 0.765; binaural beat vs. CS: *df* = 14, *t* = −2.988, *p* = 0.009, Cohen’s *d* = 0.771).

## Discussion

In this study, we proposed a novel auditory stimulus that combines binaural beat and ASMR trigger in an attempt to induce brainwave entrainment of the dominant frequency in NREM sleep stage 1. This method could reduce the inconvenience caused by binaural beat and improve the effects of brainwave entrainment. We also explored the effects of binaural beat and ASMR on brainwave entrainment and psychological stability. In session 1, it was found that combining binaural beat and ASMR trigger at a ratio of 30:60 dB was the most effective of the combinations. In session 2, four stimuli (SHAM, binaural beat, ASMR trigger, and CS) were played for 10 min, and resting states were measured for 2 min before and after each stimulus. We assumed that the effects on the previous stimulus would have almost disappeared since there were 9 to 14 min between the stimuli, including these resting states. As a result, the theta power after listening binaural beat increased over the temporal and parietal regions. There was an increased theta power over the frontal region for the ASMR trigger condition. The CS condition showed the effects of combining the ASMR trigger and the binaural beat on brainwave entrainment. The power of the theta power increased strongly in the midline associated with transition into sleep, especially after listening to CS. In regards to psychological stability using BRUMS-32, “anger” scores were clearly increased after the binaural beat condition, whereas “calmness” scores were remarkably increased after ASMR triggers and CS conditions.

It is still controversial whether the binaural beat induces specific oscillatory brainwave activity. Some studies have reported that the binaural beat with frequencies within the theta band had no effects on cognitive functions (Goodin et al., 2012; López-Caballero and Escera, 2017). One study reported no effects when the binaural beat was presented for 2 min (Goodin et al., 2012). However, this period may have been too short for cortical entrainment of these frequencies. In fact, the results of the present study suggest that at least 3 min is required to induce the effect of auditory stimuli. In addition, another study stated that the binaural beat could not be used as a potential tool for enhancing oscillatory EEG activity (López-Caballero and Escera, 2017). In their study, 373 Hz was used as the carrier tone. However, it has been reported that 250 Hz may be a better choice as a carrier of pure frequency tones (Pratt et al., 2010). Therefore, the effect of the binaural beat is not clear. However, most studies have reported that the binaural beat could activate specific brainwave frequencies and induce the desired mental states (Crespo et al., 2013; Gantt et al., 2017; Jirakittayakorn and Wongsawat, 2017). Our results clearly showed that the binaural beat induced theta brainwaves over the temporal and parietal regions. Theta power was induced, even using CS mixed with natural sounds. Auditory pathways are present in the temporal, parietal and frontal regions, which somewhat overlaps with the visual system (Hall, 2003). The primary auditory cortex is also located on the superior surface of the temporal region (Zatorre et al., 2002). Indeed, the source current density distribution of beat-evoked potentials has been shown to peak in the temporal and parietal regions (Pratt et al., 2010). Our results showed that the binaural beat-induced auditory stimuli activated the primary auditory cortex and induced a target frequency.

We observed theta power in the frontal areas for ASMR triggers. Increases in theta power in the frontal region were observed when the subjects were relaxed (Sandler et al., 2016; Zhao et al., 2018). In particular, this increase was associated with enhanced activity in the anterior cingulate cortex when in a meditative state. These changes were also thought to reflect a positive emotional state (Posner et al., 2014). Moreover, ASMR was directly involved in visceral and emotional responses by reducing the salience network associated with the dorsal anterior cingulate cortex and anterior insula (Smith et al., 2019b). In this regard, this ASMR-induced change could represent increased theta power in the frontal region. However, more research is needed into the neurophysiological mechanisms in the brain related to ASMR.

When the binaural beats combined with ASMR triggers were presented, theta power was significantly induced in all CS, regardless of the combined ratio. The individual results showed that theta power in CS2 was induced by all 15 subjects, but the four results for CS1 and three results for CS3 showed a tendency to decrease theta power. This means that the effect of binaural beat varies depending on the individual. Additionally, we investigated the spatial changes after CS. When the combined stimulus was presented, theta wave activity was most apparent in the frontal, temporal, and midline regions compared to the other brain regions. CS seemed to combine the effects of the binaural beat and the ASMR trigger. Regarding the theta power, the changes in the temporal region were prominent in CS1 with high the binaural beat, whereas the changes in the prefrontal region were prominent in CS3 with a high ASMR ratio. As a result, CS2 increased the most in the midline region associated with the transition to sleep. Similarly, in session 2, the increase in theta power in the midline was most noticeable compared with other stimuli. Previous studies suggested that the transition to sleep is marked by the increase of theta wave activity in the midline region (Wright et al., 1995; Marzano et al., 2013). Even theta power increased during unconsciousness compared to wakefulness (Lee et al., 2017). We could, therefore, speculate that the increase in theta power after CS2 in the midline region could induce sleep when combined with an auditory stimulus.

Sleep and wakefulness are controlled by an ascending arousal system that starts in the brain stem and sends projection fibers to the thalamus, hypothalamus, basal forebrain, and cerebral cortex. The system includes several nuclei groups. The nuclei of the sleep-promoting and the wake-promoting systems inhibit each other, and these changes of different neural oscillations are observed through EEG signals (Jirakittayakorn and Wongsawat, 2018). When the binaural beats enter the primary auditory cortex, the signals are transmitted directly to associated auditory areas and other relevant areas that induce the brain to oscillate at a rate of the desired frequency of binaural beats (Wahbeh et al., 2007). Specifically, these signals enter the thalamus, where audio sensory information is processed through the sensory neural pathway (Tang et al., 2015). Eventually, it is thought that auditory signals in the thalamus may affect the sleep-promoting system. In this regard, binaural beats can regulate the sleep cycle because they can be used to regulate behavioral states, followed by entrainment effects (Jirakittayakorn and Wongsawat, 2018; Perez et al., 2019). With this mechanism, our CS condition could potentially induce sleep while helping to elicit 6 Hz theta waves, which is characteristic of NREM sleep stage 1.

The effects of brain entrainment vary with the duration of stimulus exposure (Gao et al., 2014; Seifi Ala et al., 2018). In our results, the changes in alpha power were observed to be different in session 1 and session 2 with CS. There was no significant change in alpha waves in session 1, but in session 2, there was a significant decrease in alpha waves during stimulation. In session 2, the alpha power was significantly reduced for the binaural beat, ASMR trigger, and CS conditions. These results are thought to be due to different stimulus exposure times. The increase of theta power seemed to be associated with a natural decrease in alpha power as subjects enter NREM sleep stage 1 (Wright et al., 1995). From a different perspective, differences in alpha power could be linked to changes in emotion. In fact, the alpha band plays a critical role in emotional processing (Tseng et al., 2013). Previous studies reported that a decrease in the alpha band was observed after listening to natural sounds (Pineda et al., 2013). The alpha band also decreased in the left prefrontal areas when listening to positive music and in the right prefrontal areas when listening to negative music (Tseng et al., 2013; Balasubramanian et al., 2018). In other words, brain changes may be different due to the preference of the stimuli. In some cases, there has been an increase in alpha bands when listening to music (Bo et al., 2019). We observed no change in the frontal asymmetry in the alpha power related to emotion. It is thought that the preferences of the stimuli also affected individuals. Therefore, research about the relationship between alpha power and emotions would be needed to consider more precisely all variables.

For different frequencies, there were no changes in all four stimuli for delta and gamma power, but beta power significantly decreased only in the CS condition. Naturally, binaural beats and CS-induced theta power, but since delta power is the main feature in the deep NREM sleep (Lee et al., 2017; Lee M. et al., 2019) and gamma power is related to maintaining brain arousal during wakefulness (Jirakittayakorn and Wongsawat, 2018), it seems natural that these are not induced by other stimuli except for alpha power associated with emotions. Interestingly, we additionally observed the decrease of beta power in only the CS condition. Beta activity is the marker of the critical arousal (Spiegelhalder et al., 2012). In this respect, it could be considered that sleep induction caused by CS resulted in a decrease in beta power, as the NREM sleep stage 1 was reached.

As shown in the results of the BRUMS-32, ASMR trigger and CS were clearly associated with increases in positive emotions (“calmness”) and decreases in negative emotions (“anger” and “tension”). On the other hand, after listening to the binaural beat, increases in negative emotions (“anger”) and decreases in positive emotions (“happy” and “calmness”) have been revealed to be a disadvantage of the technique (Jirakittayakorn and Wongsawat, 2017). We know that the binaural beat is effective in producing the desired frequency, though it causes negative emotions. Thus, combining binaural beat with additional stimuli such as ASMR, which induces psychological stability (Barratt et al., 2017), seems to be a better way to keep the advantages of both stimuli for an effective sleep induction method.

There are several limitations to this study. First, many parameters (e.g., decibel, exposure duration, and frequency) were used to find the optimal combination of the binaural beat and ASMR trigger for inducing sleep. However, we only used three decibel ratios in this study. In future studies, efforts are needed to determine various optimal parameters. Second, we have put together a group of subjects who have heard five different ASMR triggers. Frankly speaking, because they heard different sounds, their brain responses may differ. However, in previous studies, sound listening was divided into annoying and nature sounds. These natural sounds consisted of six sounds that were similar to ours, such as river, forest, rain, jungle, ocean waves, and waterfall soundscapes. As a result, similar spatial patterns were investigated in people who listened to different natural sounds (Hong and Santosa, 2016). In this regard, we assumed that there would not be a meaningful difference in the ASMR triggers group in our study, which heard the natural sounds of different ASMR triggers. However, if we proceed with the study of the various sounds of ASMR, it would be a good opportunity to clearly investigate the changes in the brain regarding ASMR. Third, the clear evidence of sleep parameters, such as sleep onset, is a lack of sleep induction. We considered the induction of theta power, the main feature of NREM sleep stage 1, to be asleep induction, but measurements of sleep parameters are needed in the future.

## Conclusion

We investigated the effects of a CS that combined the binaural beat and ASMR trigger on two outcomes: the ability to induce brainwave entrainment and psychological stability. In order to induce sleep, it is necessary to not only induce frequencies in each sleep stage but also to be comfortable for users to reach sleep. Our proposed CS could induce the 6 Hz activity, which corresponds to the theta band, for inducing NREM sleep stage 1. In addition, the CS could be used for relieving negative emotions and increasing positive emotions for users. Our findings suggest that this could provide an effective way of improving the quality of sleep.

## Data Availability Statement

The raw data supporting the conclusions of this manuscript will be made available by the authors, without undue reservation, to any qualified researcher.

## Ethics Statement

The studies involving human participants were reviewed and approved by the Institutional Review Board at the Korea University (KUIRB-2019-0134-01). Written informed consent to participate in this study was provided by the participants’ legal guardian/next of kin.

## Author Contributions

ML, C-BS, and S-WL designed the experiments. C-BS and G-HS performed the experiments. ML, C-BS, and G-HS analyzed the data. ML and C-BS drafted the manuscript. ML and S-WL critically revised the manuscript and contributed to the important intellectual contents.

## Conflict of Interest

The authors declare that the research was conducted in the absence of any commercial or financial relationships that could be construed as a potential conflict of interest.
