# Supplementary material for: Possible Effect of Binaural Beat Combined With Autonomous Sensory Meridian Response for Inducing Sleep
Source: Front Hum Neurosci. 2019 Dec 2;13:425. doi: 10.3389/fnhum.2019.00425 (PMC6900908; doi:10.3389/fnhum.2019.00425)
Supplement: Supplementary file 1 [file Table_1.docx]

Supplementary Material

Possible Effect of Binaural Beat Combined
With Autonomous Sensory Meridian Response for Inducing Sleep

Minji Lee^1^, Chae-Bin Song^1^, Gi-Hwan Shin^1^, and Seong-Whan Lee^1, 2,*^

^1^Department of Brain and Cognitive Engineering, Korea University, Seoul, Republic of Korea
^2^Department of Artificial Intelligence, Korea University, Seoul, Republic of Korea

*** Correspondence:**Seong-Whan Lee
sw.lee@korea.ac.kr

# Supplementary Figures and Tables

## Supplementary Figures

**
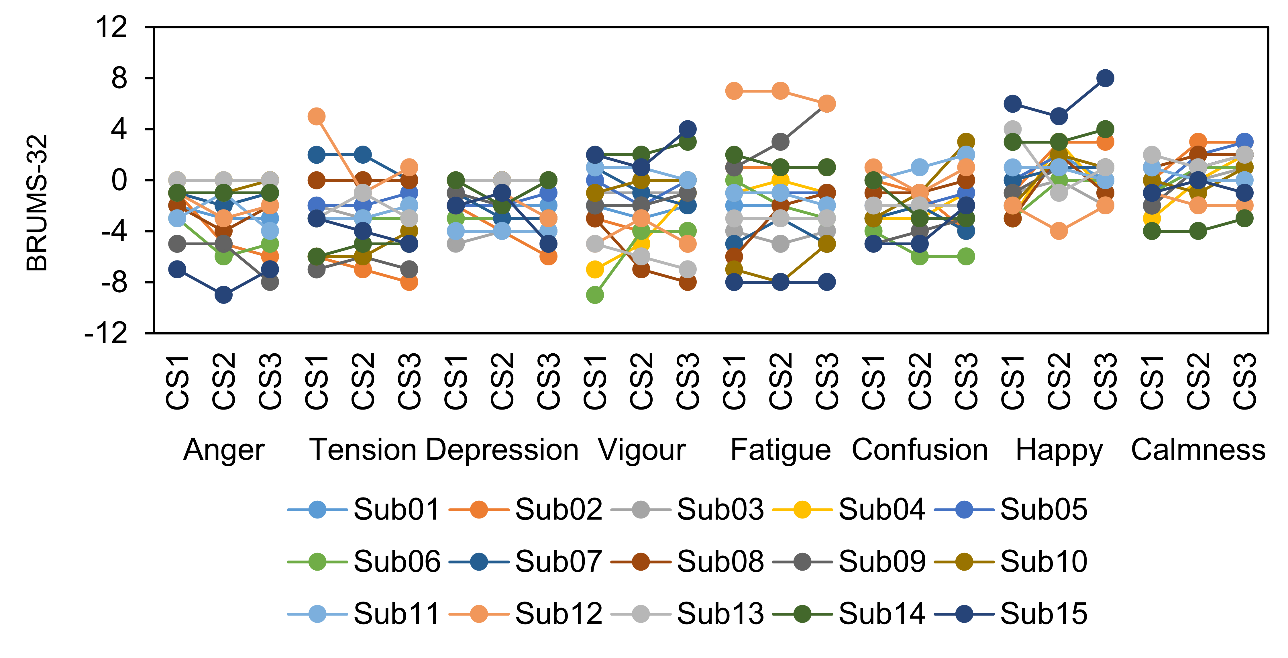
**

**Supplementary Figure 1.** **Individual changes in BRUMS-32 scores before and after the three stimulus conditions.** CS1 = 45:60 BB:AT ratio; CS2 = 30:60 BB:AT ratio; CS3 = 20:60 BB:AT ratio. BB = binaural beats, AT = autonomous sensory meridian response triggers, CS = combined stimuli of BB and AT.

**
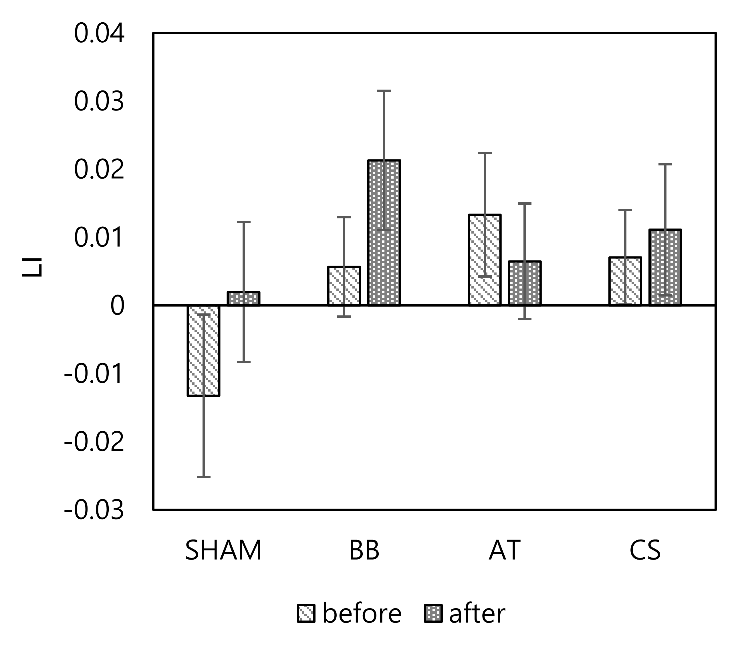
**

**Supplementary Figure 2. The alpha LI over prefrontal and frontal regions before and after four auditory stimuli in session 2.** Error bars show standard errors. SHAM = sham condition, BB = binaural beats, AT = autonomous sensory meridian response triggers, CS = combined stimuli of BB and AT at the ratio of 30:60 dB; LI = laterality index.

## Supplementary Tables

**Supplementary Table 1. The type of ASMR triggers.**

| **ASMR Triggers** | **Exposed to Subjects** |
| --- | --- |
| Rain | Sub04, Sub07, Sub14 |
| Sea waves | Sub01, Sub10, Sub13 |
| Waterfall | Sub05, Sub08, Sub11 |
| Forest | Sub02, Sub09, Sub12 |
| River | Sub03, Sub06, Sub15 |

**Supplementary Table 2. The 32-item Brunel Mood Scale.**

| **Factors** | **Descriptors** |
| --- | --- |
| Anger | Angry / Annoyed / Bad tempered / Bitter |
| Tension | Anxious / Nervous / Panicky / Worried |
| Depression | Depressed / Downhearted / Miserable / Unhappy |
| Vigour | Active / Alert / Energetic / Lively |
| Fatigue | Exhausted / Sleepy / Tired / Worn-out |
| Confusion | Confused / Uncertain / Mixed-up / Muddled |
| Happy | Cheerful / Contented / Happy / Satisfied |
| Calmness | Calm / Composed / Relaxed / Restful |

**Supplementary Table 3. Individual changes in 6 Hz peak over midline regions in session 1.** CS1 = 45:60 BB:AT ratio; CS2 = 30:60 BB:AT ratio; CS3 = 20:60 BB:AT ratio. BB = binaural beat, AT = autonomous sensory meridian responses trigger, CS = combined stimuli of BB and AT.

| **Subject** | **CS1** | **CS2** | **CS3** |
| --- | --- | --- | --- |
| **Sub01** | -0.027 | 0.188 | 0.147 |
| **Sub02** | 0.076 | 0.162 | 0.160 |
| **Sub03** | 0.102 | 0.226 | 0.221 |
| **Sub04** | -0.093 | 0.025 | -0.044 |
| **Sub05** | -0.059 | 0.001 | -0.015 |
| **Sub06** | 0.124 | 0.146 | 0.144 |
| **Sub07** | 0.162 | 0.173 | 0.158 |
| **Sub08** | 0.105 | 0.406 | 0.405 |
| **Sub09** | 0.124 | 0.178 | 0.148 |
| **Sub10** | 0.030 | 0.066 | -0.098 |
| **Sub11** | 0.580 | 0.606 | 0.416 |
| **Sub12** | -0.226 | 0.070 | 0.042 |
| **Sub13** | 0.225 | 0.317 | 0.151 |
| **Sub14** | 0.309 | 0.311 | 0.202 |
| **Sub15** | 0.236 | 0.284 | 0.212 |
| **Mean ± SD** | 0.111 ± 0.189 | 0.210 ± 0.158 | 0.150 ± 0.143 |
